# Enzyme-linked immunosorbent assay and immunohistochemical analysis of mast cell related biochemicals in oral submucous fibrosis

**DOI:** 10.12688/f1000research.141179.2

**Published:** 2024-04-29

**Authors:** Harshkant Gharote, Rahul Bhowate, Suwarna Dangore-Khasbage

**Affiliations:** 1 Oral Medicine and Radiology, Sharad Pawar Dental College and Hospital, Datta Meghe Institute of Higher Education and Reasech, Sawangi (Meghe), Wardha, Maharashtra, 442004, India

**Keywords:** Mast cells, Oral submucous fibrosis, Chymase, Histamine, Diamine oxidase

## Abstract

Oral submucous fibrosis (OSMF), a potentially malignant disorder, is developed by progressive fibrous tissue deposition in connective tissue along with atrophy of oral mucosa. Histological sections also show the mast cell infiltration in submucosa which may indicate their possible role in this entity. Abundant availability of biochemicals in mast cells like histamine and serine proteases like chymase may be released and play specific pathways in the disease pathophysiology. Possibly, if the histamine release has some part to play, diamine oxidase may also be found to have a relationship as it metabolizes histamine. The present study is proposed to identify the presence of chymase, histamine, and diamine oxidase in both, serum as well as tissue by enzyme-linked immunosorbent assay (ELISA) and immunohistochemistry (IHC) respectively. This study may provide probable insight into the mast cell-related chemicals and their association with OSMF.

## Introduction

Oral submucous fibrosis (OSMF) is a potentially malignant disorder affecting the oral cavity and oropharynx causing extensive fibrous tissue deposition in submucosa. The presence of the mast cells in histological section of OSMF has been found to be related to various stages of OSMF. Although, studies have suggested that enzymes released by degranulation of mast cells in OSMF have some role in its initiation ^1^^–^^4^ although the exact mechanism of action is not known. Therefore, it would be imperative to identify the presence of various biochemicals of mast cells in affected oral mucosa and perhaps the serum of the affected individuals. Hence, it can be stated that if there is histamine release during the initiation and progression of OSMF, possible increased levels of serum histamine levels can be observed in those patients. Additionally, in response to increased histamine levels, its metabolism will be initiated which is mediated through enzyme diamine oxidase (DAO). Therefore, variation in serum DAO levels may also be expected in those individuals.

Mast cells (MCs) are multifaceted immune cells subclassified based on their protease content as mast cell with tryptase only (MCT) and mast cell with tryptase and chymase (MCTC) and may have varied role in various pathogeneses including cancer. Mast cells exhibit increased accumulation within tumor microenvironments and attribute to prognosis, metastasis and reduced survival in several types of human cancer. mast cells can influence the tumor microenvironment and induce pro-tumor effects by conditioning the fate of tumor cells, initiation of angiogenesis and tissue remodeling though its bioactive molecules. including drug resistance. ^5^^–^^7^ Further, OSMF shows high potential for malignant transformation and is correlated to a series of biochemical alterations seen during the progress of the disease. The incidence of mast cells in the affected mucosa may be implicated in the malignant transformation. Studies show that mast cell serine proteases like tryptase and chymase significantly impact angiogenesis thus affecting tumor development and progression. ^8^ There is evidence that supports an association between mast cell chymase and tumor angiogenesis. While tryptase, a neutral serine protease, is the abundant mediator stored in the mast cell granules that mediate degranulation of mast cells in allergic diseases. ^9^ Thus, among these, evaluation of serum chymase may provide insights into the function of mast cells in the initiation and progression of OSMF.

## Protocol

The present study proposes that variations in the serum levels of diamine oxidase, histamine, and chymase may be noticed in OSMF. With this view, the present study will attempt to estimate the serum levels of chymase, histamine, and diamine oxidase in various stages of OSMF and compare them with the levels in healthy individuals and persons with areca habit without OSMF. Additionally, an immunohistochemical analysis will be performed for mast cell-related markers- chymase, diamine oxidase, and histamine to extrapolate their presence in serum.

The present research study has been approved by the Institutional Ethical Committee and will be conducted in the Department of Oral Medicine and Radiology, Sharad Pawar Dental College and Hospital, Wardha, India.

***Aim:*** Assessment of variations in serum diamine oxidase, histamine, and chymase levels in the various stages of OSMF, areca chewers without OSMF, and healthy individuals by an enzyme-linked immunosorbent assay.

## Objectives

1. To estimate serum histamine and diamine oxidase levels in the various stages of OSMF and between overall OSMF patients, areca chewers without OSMF, and healthy individuals.
2. To estimate serum chymase levels in the various stages of OSMF and between overall OSMF patients, areca chewers without OSMF, and healthy individuals.
3. To compare serum histamine and diamine oxidase levels in various stages of OSMF and between overall OSMF patients, areca chewers without OSMF, and healthy individuals.
4. To compare serum chymase levels in the various stages of OSMF, and between overall OSMF patients, areca chewers without OSMF, and healthy individuals.
5. To correlate serum diamine oxidase levels with serum histamine levels in the various stages of OSMF and between overall OSMF patients, areca chewers without OSMF, and healthy individuals.
6. To correlate serum histamine, chymase, and diamine oxidase levels in various stages of OSMF and between overall OSMF patients, areca chewers without OSMF, and healthy individuals.
7. To analyze the immunohistochemical expression of mast cell chymase, histamine, and diamine oxidase in OSMF, patients with areca habit without OSMF, and healthy individuals.
8. The serum values will be correlated to the immunohistochemistry expressions of the chymase, diamine oxidase and histamine to identify the possible relationship.

## Method

### Experimental design

The participants will be divided into three groups as OSMF group, individuals with areca habit without OSMF group, and Healthy individuals’ group. All individuals included will be above 18 years without any systemic or metabolic disorder. OSMF patients with a history of treatment will be excluded from the study. Written consent will be obtained after an explanation of the study procedure and protocol for the collection of serum samples and biopsy specimens.

### Selection of OSMF patients

Individuals with OSMF will be selected following functional staging classification of proposed by More et al. ^10^ The classification of functional stages is given as follows:

Functional staging:

‐ M1: Interincisal mouth opening - up to or greater than 35 mm.
‐ M2: Interincisal mouth opening between 25 and 35 mm.
‐ M3: Interincisal mouth opening between 15 and 25 mm.
‐ M4: Interincisal mouth opening less than 15 mm.

### Selection of individuals with areca habit without OSMF

Individuals with a history of areca consumption of more than one year duration, without any evidence of OSMF and other oral mucosal conditions like leukoplakia and lichen planus; and without history of any medical disorders

### Selection of Healthy individuals

Individuals without OSMF and without habits and any systemic disorders/conditions.

The details of selected participants will be recorded in the case history proforma for recording clinical findings and investigations. The informed consent will be obtained before enrolling the participant for proposed investigations.

### Exclusion criteria

1. The individuals with a history of the consumption of tobacco in any other form such as cigarette, bidi, and tobacco with lime.
2. The individuals with a history of any systemic disease.
3. The patients having history of receiving treatment for OSMF.
4. The patients having history for antihistaminic medications.

The proposed study, a case control study, will include OSMF patients, areca habitual without OSMF and healthy individuals. The population of Indian subcontinent is infinite, and the individuals affected by OSMF are variable from state to state, therefore no definite data is available. Yet, the latest published literature shows that the prevalence of OSMF in India is 7.21%, ^11^ the sample size can be calculated using the formula for known population proportion (p). The sample size selection will be at 95 per cent confidence interval with margin of error (d) at ±10%. Thus, following formula can be used to calculate sample size:

$$n=\frac{z^{2}p\left(1-p\right)}{d^{2}}$$

In the formula:

n = sample size

z = z score (1.96)

p = population proportion (0.0721)

d = margin of error (0.1)

$$n=\frac{{\left(1.96\right)}^{2}0.0721\left(1-0.0721\right)}{0.1^{2}}$$

n = 25.7

Thus, 26 individuals can be enrolled in each group for the study. Thus, a total sample size of 78 will be used for balanced study with each group consisting of 26 subjects.

Five histological sections each for overall OSMF patients, areca chewers without OSMF and healthy individuals will be subjected for immunohistochemical analysis. Thus, 15 histopathological slides will be subjected to immunohistochemical expression for each marker viz. histamine, diamine oxidase and chymase.

### Materials

Commercially available ELISA kits will be procured for the estimation of serum histamine, chymase and diamine oxidase. The optical density will be measured by spectrophotometry at a wavelength of 450 nm ± 2 nm using an ELISA reader. The immunohistochemical analysis will be performed on formalin-fixed paraffin-embedded biopsy tissue sections of OSMF patients.

### Procedure

***Collection of serum samples:*** Blood sample will be drawn from antecubital vein using a 5 ml syringe and it will be taken into a vial containing a clot activator and serum gel separator. After that, it will be transferred into a centrifuge tube. The centrifugation of blood will be done for ten minutes, and serum will be obtained. Centrifugation is a procedure of using centrifugal force and the aim is to separate two unmixable liquids. There is sedimentation of heterogeneous mixtures in which more heavy constituents drift away from the axis of centrifuge and less heavy constituents drift towards the axis of centrifuge. The effective gravitational force on a test tube is increased which causes the precipitate to collect on the bottom of tube and the remaining supernatant liquid will be withdrawn with the help of a pipette.

***Estimation of serum Diamine oxidase levels:*** This ELISA kit will use the Sandwich-ELISA principle. The ELISA plate provided is pre-coated with an antibody specific to Mouse DAO. Standards or samples will be added to the ELISA plate wells and combined with the specific antibody. Next, a biotinylated detection antibody specific for Mouse DAO and Avidin-Horseradish Peroxidase (HRP) conjugate will be added sequentially to each micro plate well and incubated. Free components are washed away. The substrate solution will be added to each well. Only those wells that contain Mouse DAO, biotinylated detection antibody and Avidin-HRP conjugate will appear blue in color. The enzyme-substrate reaction will be terminated by the addition of stop solution and the color turns yellow. The optical density (OD) will be measured using spectrophotometry at a wavelength of 450 nm ± 2 nm. The OD value will be proportional to the concentration of Mouse DAO. The concentration of Mouse DAO in the samples will be calculated by comparing the OD of the samples to the standard curve.

***Estimation of serum Histamine levels:*** After Histamine is quantitatively acylated, the subsequent competitive ELISA kit will be used the microtiter plate format with the antigen bound to the solid phase. The acylated standards, controls and samples and the solid phase bound analyte will compete for a fixed number of antiserum binding sites. After the system is in equilibrium, free antigen and free antigen-antiserum complexes will be removed by washing. The antibody bound to the solid phase will be detected by an anti-rabbit IgG peroxidase conjugate using 3,3′,5,5′-tetramethylbenzidine (TMB) as a substrate. The reaction will be monitored at 450 nm. Quantification of unknown samples will be achieved by comparing their absorbance with a reference curve prepared with known standard concentrations.

***Estimation of serum chymase levels:*** This ELISA kit will use the Sandwich ELISA principle. The ELISA plate provided in this kit is precoated with an antibody specific to Human CMA1. Standards or samples will be added to the ELISA plate wells and combined with the specific antibody. Then, a biotinylated detection antibody specific for Human CMA1 and Avidin Horseradish Peroxidase (HRP) conjugate will be added successively to each micro plate well and incubated. Free components will be washed away. The substrate solution will be added to each well. Only those wells that contain Human CMA1, biotinylated detection antibody and Avidin HRP conjugate will appear blue in color. The enzyme substrate reaction will be terminated by the addition of stop solution and the color turns yellow. The optical density (OD) will be measured spectrophotometrically at a wavelength of 450 nm ± 2 nm. The OD value will be proportional to the concentration of Human CMA1. The concentration of Human CMA1 in the samples will be calculated by comparing the OD of the samples to the standard curve.

***Immunohistochemistry:*** The tissue sections will be deparaffinized in xylene solution and rehydrated through decreasing graded ethanol solution. Endogenous peroxidase activity will be inhibited by incubation for 10 minutes with 3% hydrogen peroxidase. The primary monoclonal antibodies used for mast cells will be anti-MC chymase, anti-MC diamine oxidase, and antihistamine antibodies. Staining will be performed at room temperature on an automatic staining workstation. Commercially available immunohistochemistry kits will be procured for these markers.

### Statistical analysis

The statistical calculations will be performed by using following tests:

1. Students t-test
2. Analysis of variance (ANOVA)
3. Pearson’s correlation

## Expected outcomes

As numerous mast cells are found to be present histologically in OSMF, it is expected to see the possible increase in serum levels of diamine oxidase, histamine, and chymase. Further, the presence of these enzymes will be confirmed by immunohistochemical analysis of histological sections. positive results in both the methods will give an insight into the role of these enzymes in progression and the possible role in the malignant transformation of OSMF.

If the outcomes reveal statistically nonsignificant variation in serum levels of these biomolecules, role of mast cells in systemically influencing the initiation and progression of OSMF will be ruled out. Nonetheless, increased serum levels might be indicative of the active secretory action of mast cells and may provide an understanding of their role in progression of OSMF in oropharynx and esophagus. Tissue expression of histamine and chymase will establish a possible role of histamine receptors and chymase in induction of fibrosis and futuristic approach to malignant transformation of the OSMF.

## Discussion

The prevalence of OSMF in India is 7.21% and shows higher rates of malignant transformation. ^11^ The presence of mast cells in histological sections reveal their strong association with the disease so that some biochemicals and enzymes might play role in the progression and malignant transformation. ^3^^,^^12^ Thus, detection of the mast cell-related chemicals in tissue and serum will delineate further mechanisms in pathogenesis. With special emphasis on diamine oxidase and its relation to histamine, the possible allergic response of mast cells to etiological factors like betel nut will be discerned. Further, possible detection of chymase in serum will define its elaborate role in local as well as a systemic mechanism in angiogenesis in OSMF.

Infiltration of mast cells in OSMF and other premalignant disorders like lichen planus and leukoplakia has been studied and are implicated in initiation and progression of these entities. ^4^^,^^13^^,^^14^ The studies are conducted on expression of MCT and MCTC in OSMF using immunohistochemistry and observed a significant increase in expression of MCTC in OSMF. ^15^ Further, serum histamine and tryptase levels have been assessed in oral squamous cell carcinoma (OSCC) along with its correlation with various histological zones/stages. While histamine was positively correlated to the depth of the invasion, serum tryptase had no correlation with various grades of OSCC. ^16^^,^^17^ Moreover, as mast cells are associated with collage metabolic disorders, it is suggested that they play similar role in pathogenesis of OSMF and labelled it as a collagen metabolic disorder. ^18^ Nonetheless, the literature gap shows need to understand the role of chymase and histamine in pathogenesis of OSMF and its malignant transformation. Further, mast cell related research on their degranulation and release of bioactive molecules may provide an insight to understand the pathophysiology of OSMF as a collagen metabolic disorder.

### Ethical considerations

The present study is approved by the Institutional Ethics Committee of Datta Meghe Institute of Higher Education and Research, Sawangi Wardha, India with the ethical approval reference no. DMIMS (DU)/Ph.D. Regn. /2021/1226.

### Study status

The present study is in the process of data collection wherein serum samples are being collected from OSMF group, Areca habitual and control groups as per the design.

## Data Availability

Not applicable as it is a proposed protocol.

## References

[1] Sirsat SM Pindborg JJ : Mast cell response in early and advanced oral submucous fibrosis. *Acta Pathol. Microbiol. Scand.* 1967;70:174–178. 6050370 10.1111/j.1699-0463.1967.tb01279.x

[2] Pujari R Vidya N : Mast cell density in oral submucous fibrosis: a possible role in pathogenesis. *Int. J. Health Sci (Qassim).* 2013 Jan;7(1):23–29. 10.12816/0006017 23559902 PMC3612412

[3] Kumar LB Mathew P Madhavan N : Evaluation of mast cells and burning sensation in various stages of Oral Submucous Fibrosis. *J. Oral Biol. Craniofac. Res.* 2020 Oct-Dec;10(4):430–434. 10.1016/j.jobcr.2020.07.005 32793410 PMC7414006

[4] Ankle MR Kale AD Nayak R : Mast cells are increased in leukoplakia, oral submucous fibrosis, oral lichen planus and oral squamous cell carcinoma. *J. Oral Maxillofac. Pathol.* 2007;11:18–22. 10.4103/0973-029X.33959

[5] Zhao XO Sommerhoff CP Paivandy A : Mast cell chymase regulates extracellular matrix remodeling-related events in primary human small airway epithelial cells. J. Allergy Clin. Immunol. 2022 Dec;150(6):1534–1544. Epub 2022 Jun 30. 10.1016/j.jaci.2022.05.028 35779668

[6] Aponte-López A Fuentes-Pananá EM Cortes-Muñoz D : Mast Cell, the Neglected Member of the Tumor Microenvironment: Role in Breast Cancer. *J. Immunol. Res.* 2018 Feb;2018:2584243. 10.1155/2018/2584243 29651440 PMC5832101

[7] Maciel TT Moura IC Hermine O : The role of mast cells in cancers. *F1000Prime Rep.* 2015 Jan 5;7:09. 10.12703/P7-09 25705392 PMC4311277

[8] De Souza Junior DA Santana AC Silva EZM da : The Role of Mast Cell Specific Chymases and Tryptases in Tumor Angiogenesis. *Biomed. Res. Int.* 2015;2015:1–13. 10.1155/2015/142359 PMC4471246 26146612

[9] Mahmood M Rashid BM Amin K : Correlation between Serum Tryptase Level and Disease Severity in Asthmatic Patients in the Sulaimani Governorate. *Int. J. Med. Res. Health Sci.* 2016;2016(5):34–41.

[10] More CB Gupta S Joshi J : Classification System for Oral Submucous Fibrosis. *J. Indian Aca. Oral Med. Radiol.* 2012;24(1):24–29. 10.5005/jp-journals-10011-1254

[11] Rao NR Villa A More CB : Oral submucous fibrosis: a contemporary narrative review with a proposed inter-professional approach for an early diagnosis and clinical management. *J. Otolaryngol. Head Neck Surg.* 2020 Jan 8;49(1):3. 10.1186/s40463-020-0399-7 31915073 PMC6951010

[12] Agostinelli E Tempera G Viceconte N : Potential anticancer application of polyamine oxidation products formed by amine oxidase: a new therapeutic approach. *Amino Acids.* 2010 Feb;38(2):353–368. 10.1007/s00726-009-0431-8 20012114

[13] Kinra M Ramalingam K Sarkar A : Comparison of mast cell count and mast cell densit y in normal mucosa, oral leukoplakia, oral lichen planus, oral submucous fibrosis and oral squamous cell carcinoma - a study of 50 cases. *JPSI.* 2012;1:4–11.

[14] Sundararajan A Muthusamy R Gopal Siva K : Correlation of Mast Cell and Angiogenesis in Oral Lichen Planus, Dysplasia (Leukoplakia), and Oral Squamous Cell Carcinoma. *Rambam Maimonides Med. J.* 2021 Apr 29;12(2): e0016. 10.5041/RMMJ.10438 33938803 PMC8092953

[15] Yadav A Desai RS Bhuta BA : Altered immunohistochemical expression of mast cell tryptase and chymase in the pathogenesis of oral submucous fibrosis and malignant transformation of the overlying epithelium. *PLoS One.* 2014 May 29;9(5): e98719. 10.1371/journal.pone.0098719 24874976 PMC4038611

[16] Hasan NR Dehuri P Jena A : A Correlation of Serum Histamine and Mast Cell Count with the Established Prognosticators in Oral Cancer. *J. Microsc. Ultrastruct.* 2023 Jan 5;11(2):97–102. 10.4103/jmau.jmau_138_20 37448818 PMC10337676

[17] Jaafari-Ashkavandi Z Khademi B Akbari S : Serum level of mast cell tryptase in patients with oral squamous cell carcinoma: lack of correlation with clinicopathologic factors. *Asian Pac. J. Cancer Prev.* 2013;14(5):2955–2958. 10.7314/apjcp.2013.14.5.2955 23803060

[18] Rajalalitha P Vali S : Molecular pathogenesis of oral submucous fibrosis--a collagen metabolic disorder. *J. Oral Pathol. Med.* 2005 Jul;34(6):321–328. 10.1111/j.1600-0714.2005.00325.x 15946178

