# Supplementary material for: Enzyme-linked immunosorbent assay and immunohistochemical analysis of mast cell related biochemicals in oral submucous fibrosis
Source: F1000Res. 2024 May 20;12:1288. Originally published 2023 Oct 9. [Version 3] doi: 10.12688/f1000research.141179.3 (PMC11140300; doi:10.12688/f1000research.141179.3)
Supplement: Peer review history

# Reviewer report: Reviewer response for version 3

**DOI:** 10.5256/f1000research.166384.r280429

**Published:** 2024-05-30

**Authors:** Vini Mehta

**Affiliations:** 1 Dr. D. Y. Patil Dental College and Hospital, Pimpri, India

The authors have satisfactorily done all the corrections. 

 The manuscript can be indexed in the current form.

Is the study design appropriate for the research question?

Yes

Is the rationale for, and objectives of, the study clearly described?

Yes

Are sufficient details of the methods provided to allow replication by others?

Yes

Are the datasets clearly presented in a useable and accessible format?

Yes

Reviewer Expertise:

Public Health, Oral Cancer,

I confirm that I have read this submission and believe that I have an appropriate level of expertise to confirm that it is of an acceptable scientific standard.

# Reviewer report: Reviewer response for version 2

**DOI:** 10.5256/f1000research.165338.r270235

**Published:** 2024-05-10

**Authors:** Vini Mehta

**Affiliations:** 1 Dr. D. Y. Patil Dental College and Hospital, Pimpri, India

1. Please club the objectives. 

 2. Use software for sample size estimation. 

 3. Will CTRI registration be done for this study? 

 4. Write which software will be used for statistical analysis? which version? Complete the statement regarding confidence interval and p values. 

 5. Add the following reference: 

 - Mast cell mediated myofibroblast differentiation responsible for progression of oral submucous fibrosis

Is the study design appropriate for the research question?

Yes

Is the rationale for, and objectives of, the study clearly described?

Yes

Are sufficient details of the methods provided to allow replication by others?

Yes

Are the datasets clearly presented in a useable and accessible format?

Yes

Reviewer Expertise:

Public Health

I confirm that I have read this submission and believe that I have an appropriate level of expertise to confirm that it is of an acceptable scientific standard, however I have significant reservations, as outlined above.

# Reviewer report: Reviewer response for version 2

**DOI:** 10.5256/f1000research.165338.r270102

**Published:** 2024-05-07

**Authors:** Xinjia Cai

**Affiliations:** 1 Peking University School and Hospital of Stomatology, Beijing, China

There are no further comments.

Is the study design appropriate for the research question?

Yes

Is the rationale for, and objectives of, the study clearly described?

Partly

Are sufficient details of the methods provided to allow replication by others?

Yes

Are the datasets clearly presented in a useable and accessible format?

Not applicable

Reviewer Expertise:

Malignant transformation of oral potentially malignant disorders.

I confirm that I have read this submission and believe that I have an appropriate level of expertise to confirm that it is of an acceptable scientific standard.

# Reviewer report: Reviewer response for version 1

**DOI:** 10.5256/f1000research.154596.r254085

**Published:** 2024-04-01

**Authors:** Xinjia Cai

**Affiliations:** 1 Peking University School and Hospital of Stomatology, Beijing, China

This manuscript provides a study protocol for enzyme-linked immunosorbent assay and immunohistochemical analysis of mast cell-related biochemicals in OSMF, and although some plans for the experiments are provided, the details of the experimental methodology and subsequent analyses need to be further optimized, and the Discussion section of the article should have included a discussion of studies on mast cells and OSMF or oral potentially malignant disorders, which would have enabled the reader to understand the potential significance of the study.

 However, studies related to mast cells in OSMF deserve to be investigated. Mast cells are myeloid-derived immune cells located primarily in tissues in contact with the external environment that can be stimulated by a variety of different triggers to act as sentinel immune cells, recruiting other immune cells into the affected microenvironment and altering their function by releasing chemokines, cytokines and other factors. The study of mast cells might help to elucidate the changes in the immune microenvironment in OSMF development and malignant transformation and provide an experimental basis for the development of immunotherapies.

 Introduction:

 Mast cells could be classified into two groups according to whether they contain tryptase without chymase (MC _T_) or both tryptase and chymase (MC _TC_), and according to the studies that have been reported, there were different roles for the two mast cell subtypes in cancer progression, and it is possible that both tryptase and chymase might play a role in the developments and malignant transformation of OSMF.

 Objectives:

 Since OSMF is a disease confined to the oral mucosa, it is suggested to correlate the serum levels and immunohistochemical expression levels of the mast cell secretions to clarify whether the changes in immunohistochemical expression levels would be more pronounced than the changes in serum levels.

 As mast cells can be divided into two subtypes, MC _T_ and MC _TC_, the analysis of changes in mast cell subtypes and their spatial localization using immunofluorescence could be one of the further progress in this study.

 Method:

 How was the P (population ratio) of 0.0721 in the sample size calculation determined and is it based on any references?

 Please describe the tools or methods used to identify molecular expression after immunohistochemical staining of tissue sections.

 Expected outcomes:

 The effect of mast cells on the malignant transformation cannot be identified in the current study protocol. The prognostic information of the patient needs to be clarified (e.g. through regular clinical examination or follow-up, etc.), and correlation analyses between molecular expression and prognostic information need to be included in the study objectives, which may help to understand the immune mechanisms and microenvironmental alterations of malignant transformation in OSMF.

 Discussion: The current Discussion section does not provide enough information. Studies have reported the role of mast cells in OSMF as well as in other oral potentially malignant disorders and oral cancer, and although this study was only a study protocol, the authors should have discussed the results of the previous studies, clarifying the differences between this study and the previous studies, the existence of any additions to the previous results, and the implications of the study in terms of inspiration for future research. In the current manuscript, understanding the potential mechanisms of mast cell-related biochemicals in OSMF pathogenesis or carcinogenesis is difficult, and deepening the Discussion section may help to remedy this.

Is the study design appropriate for the research question?

Yes

Is the rationale for, and objectives of, the study clearly described?

Partly

Are sufficient details of the methods provided to allow replication by others?

Yes

Are the datasets clearly presented in a useable and accessible format?

Not applicable

Reviewer Expertise:

Malignant transformation of oral potentially malignant disorders.

I confirm that I have read this submission and believe that I have an appropriate level of expertise to confirm that it is of an acceptable scientific standard, however I have significant reservations, as outlined above.

# Response

**Published:** 2024-04-19

**Authors:** Harshkant Gharote

Suitable changes are made with addition of relevant information in the introduction section as suggested.

 Sample size calculation is elaborated with citation.  

 Expected outcomes have been explained in details in context to the results of the assays.

 Discussion is supported with other studies with citations and the relevant information.
